# Does Herbal and/or Zinc Dietary Supplementation Improve the Antioxidant and Mineral Status of Lambs with Parasite Infection?

**DOI:** 10.3390/antiox9121172

**Published:** 2020-11-24

**Authors:** Klaudia Čobanová, Zora Váradyová, Ľubomíra Grešáková, Katarína Kucková, Dominika Mravčáková, Marián Várady

**Affiliations:** 1 Centre of Biosciences of the Slovak Academy of Sciences, Institute of Animal Physiology, 040 01 Košice, Slovakia; varadyz@saske.sk (Z.V.); gresakl@saske.sk (Ľ.G.); kuckova@saske.sk (K.K.); mravcakova@saske.sk (D.M.); 2 Institute of Parasitology, Slovak Academy of Sciences, 040 01 Košice, Slovakia

**Keywords:** herbal treatment, organic zinc, lamb, *Haemonchus contortus*, antioxidant enzymes, lipid peroxidation, mineral status

## Abstract

This study was conducted to evaluate the effect of feed supplementation with a medicinal herbs mixture (Hmix) and organic zinc (Zn), alone or in combination, on the antioxidant responses and mineral status of lambs infected with the gastrointestinal nematode parasite *Haemonchus contortus*. A total of 24 experimentally infected lambs were randomly allocated to 1 of 4 dietary treatments (*n* = 6). The diets included an unsupplemented control diet (CON) and the CON further supplemented with Hmix, Zn, or both Hmix + Zn. Antioxidant enzymes activities, lipid peroxidation, total antioxidant capacity (TAC) and microelement (Zn, Cu, Fe, Mn) concentrations were analyzed in serum, liver, kidney, and intestinal mucosa. Zinc treatment elevated the superoxide dismutase activities in the duodenal mucosa and ileal TAC. Intake of Hmix resulted in higher kidney and ileal catalase activity and also influenced the TAC of the liver and intestinal mucosa. The inclusion of Hmix or Zn alone into the diet increased glutathione peroxidase activity in the blood, liver and duodenal mucosa. Tissue mineral uptake was not affected by herbal supplementation. Organic Zn intake increased the serum and liver Zn levels and influenced the Cu concentration in duodenal mucosa. Dietary supplementation with Hmix and/or Zn might promote the antioxidant status of lambs infected with *Haemonchus* spp.

## 1. Introduction

Parasitic gastrointestinal nematodes lead to an adverse impact on health and productivity in livestock, thus causing huge economic losses worldwide. The blood-sucking parasite *Haemonchus contortus* is in particular the predominant pathogenic endoparasite of small ruminants [1]. Nowadays, various trace elements or herbal nutraceuticals which have anthelmintic properties and can be successfully serve as an alternative to chemical control of parasites have been identified [2,3,4].

The presence of the parasite may induce the formation of a large number of reactive molecules derived from oxygen, such as superoxide radicals, hydroxyl radicals, and hydrogen peroxide [5]. The production of reactive species by phagocytes is a defensive mechanism of the host against the parasites but it promotes oxidative stress, which may also cause serious tissue damage to animals when an imbalance between free radicals’ formation and the capacity of the antioxidant defense system occurs [5,6]. The use of medicinal herbs containing bioactive compounds and trace elements may boost a host’s antioxidant response to combat oxidative stress and prevent or attenuate the pathological transition caused by parasitic infection [2,7,8].

Due to their redox properties and chemical structure, flavonoids and other phenolic compounds exert antioxidant activity by means of several mechanisms precisely described mainly in vitro [9]. However, the antioxidant effect of polyphenols/flavonoids in biological systems may depend on the efficiency of their absorption and extensive metabolic transformation, the active concentration of flavonoids and their metabolites accumulated in the target tissues as well as on surrounding chemical environment, which varies according to tissues and physiological conditions [10]. For these reasons, the effect of medicinal herbs, rich in plant secondary metabolites with bioactive properties, on the gut antioxidant status and animal health in general needs further elucidation.

Several trace elements are integral components of various antioxidant enzymes and play an essential role as components of antioxidant defense against free radical-induced tissue damage [11]. The presence of gastrointestinal nematode infection can seriously disturb the mineral metabolism of ruminants. In haemonchosis, iron metabolism can be strongly affected, particularly due to anemia in infected animals, and parasitism has a depressive effect on blood copper level in sheep [4,12]. Microelements, such as zinc, copper, and selenium, are essential for the development of functional immunity against parasites [13].

Zinc belongs to the essential trace elements that act as a co-factor for enzymes important for ensuring the proper functioning of the antioxidant defense system. Physiological or adequate levels of zinc protect cells against oxidative damage through the stabilization of cell membranes, inhibition of pro-oxidant enzymes, induction of antioxidant system response and metallothionein synthesis, all of which play a central role in oxidoreductive cellular metabolism [14,15]. Pivoto et al. [7] reported that zinc edetate administered subcutaneously could contribute to the alleviation of the oxidative stress induced by *H. contortus* in lambs.

Some researchers have reported that supplementation with organic Zn sources (amino acid complexes) may improve the growth rate of lambs and increase apparent absorption and retention of zinc, indicating higher bioavailability from organic sources also due to higher zinc concentration in serum and tissues as compared to inorganic source [16,17]. Van Valin et al. [18] found similar zinc retention in lambs regardless of the supplemental Zn source or dietary Zn concentration and pointed out the importance of elucidating dietary factors affecting zinc bioavailability in ruminants. Several studies concerning the interactions between various bioactive plant compounds and mineral absorption in the intestine or their deposition have been reported, with different results depending on the polyphenols used and the microelement examined [19,20]. Plant polyphenolic compounds can form complexes with metal ions which are stable over a wide pH range and throughout the entire gastrointestinal tract but which can inhibit mineral absorption and their bioavailability in ruminants [21].

Information on the antioxidant response of lambs suffering from nematode infection to feed supplementation with herbal nutraceuticals and/or zinc has thus been limited, and to the best of our knowledge, no studies have been undertaken to examine whether the use of dietary additives alone or in combination can affect microelements tissue uptake. Considering the occurrence of oxidative stress in lambs experimentally infected with the gastrointestinal nematode *H. contortus*, this article aims to assess the efficacy of medicinal herbs and organic zinc in improving animal antioxidant status and in influencing microelement (Zn, Cu, Fe, and Mn) levels in the liver, kidney, and intestinal mucosa.

## 2. Materials and Methods

### 2.1. Experimental Design and Feeding

All animals were handled in accordance with the guidelines for animal experiments set out in the European Community Directive (2010/63/EU). The study design and all procedures were approved by the Ethics Committee of the Institute of Parasitology of the Slovak Academy of Sciences in accordance with national legislation in Slovakia—Animal Welfare Act No. 23/2009. The animals used in this study were part of comprehensive research aimed at the impact of herbal nutraceuticals and/or zinc for controlling haemonchosis in experimentally infected lambs, and some parts, including the experimental conditions, have been previously described in detail [22].

A total of 24 female lambs (Improved Valachian), 3–4 months old and weighing an average 15.12 ± 1.58 kg, were housed in common stalls with free access to water. At the onset of the experiment, the animals had 7 days to adapt to the environment and consumed meadow hay (ad libitum) and the same commercial concentrate (500 g dry matter (DM)/day/animal). The concentrate was composed of 370 g/kg of wheat bran, 200 g/kg of soybean meal, 230 g/kg of rolled oats and 200 g/kg of maize meal, and the daily ration was divided into two portions offered at ca. 7:00 AM and 3:00 PM. The chemical composition of the meadow hay, concentrate (with and without Zn supplement) and herbal mix are summarized in Table 1. After the adaptation period, all animals were checked for parasite infection using the McMaster method [23]. Subsequently, parasite-free lambs were experimentally infected with approximately 5000 third-stage larvae of *Haemonchus contortus* administered orally to each lamb. For infection, we used the MHco1 strain of *H. contortus*, which is susceptible to all the main classes of anthelmintics. The infection was successful in all infected animals, as was previously shown by Váradyová et al. [22]. Thereafter, all infected lambs were randomly assigned to 1 of 4 dietary treatment groups of six animals in each. The number of animals used in the experiment was assigned according to VICH GL13 guidelines proposed by the European Medicines Agency. The diets included the unsupplemented control diet (CON) and this diet further supplemented with either a Herbal mixture (Hmix), Zn-chelate of glycine hydrate (Zn), or both additives (Hmix + Zn). The animals were housed in collective stalls according to the dietary treatment (*n* = 6/stall) and the experimental period lasted 70 days (during summer).

The herbal mixture (Hmix) used in our study was a non-commercial product composed of herbs typical for Central Europe, which were chosen based on the information about their phytotherapeutic properties from traditional ethnomedicine practice. The Hmix was prepared as a mixture of 9 different dry herbs obtained from commercial sources (AGROKARPATY, Plavnica, Slovak Republic, and BYLINY Mikeš s.r.o, Čičenice, Czech Republic). The mix of medicinal herbs consisted of 11.8% each of *Althaea officinalis* L. (root), *Petasites hybridus* L. (root), *Inula helenium* L. (root), *Plantago lanceolata* L. (leaf), *Rosmarinus officinalis* L. (leaf), *Solidago virgaurea* L. (stem), *Fumaria officinalis* L. (stem), *Hyssopus officinalis* L. (stem), and 5.6% *Foeniculum vulgare* Mill. (seed). Quantitative analysis of the bioactive compounds in Hmix identified three main groups: Flavonoids (54%) with a high concentration of quercetin, verbascoside and luteolin; diterpenes (27%) with a high concentration of carnosic acid and carnosol; and phenolic acids (19%) with a high concentration of rosmarinic acid and chlorogenic acid [22]. The lambs were fed the Hmix in the amount of 100 g DM/day/animal which was mixed daily with commercial concentrate (with or without Zn supplement) during the experimental period.

The organic zinc source (Zn-chelate of glycine hydrate, Glycinoplex-Zn 26%; Phytobiotics Futterzusatzstoffe GmbH, Eltville, Germany) was directly mixed with the commercial concentrate to provide an additional 60 mg Zn/kg and the concentration was analytically confirmed in triplicates (86.0 mg Zn/kg DM). Consumption of the experimental diets was visually controlled after each feeding and no dietary refusal was recorded. Throughout the whole experiment, the animals had free access to fresh potable water and a specially-prepared mineral lick without zinc offered to each lamb once a week. The mineral lick consisted of (g/kg) 16.2 Ca, 316 Na, 32 Mg, 0.7 Cu, 2.5 Mn, 0.06 Co, 0.02 I, and 0.01 Se.

### 2.2. Sample Collection

At the end of the experiment, the jugular blood samples were collected from fasting lambs into heparinized tubes in the early morning, just before the sacrifice of each animal. Serum samples were obtained from blood collected into 10-mL serum-separate tubes (Sarstedt AG &Co, Nümbrecht, Germany) and the serum was then separated by centrifugation at 1200× *g* for 10 min at room temperature. The blood and sera samples were stored at −80 °C until analysis. All animals were euthanized (abattoir of the Centre of Biosciences of SAS, Institute of Animal Physiology, Košice, Slovakia, No. SK U 06018) using an overdose of pentobarbital (Dolethal, Vetoquinol, Towcester, Northamptonshire UK). The samples of liver, kidneys and small intestine were excised immediately after death. The duodenal, jejunal and ileal sections were thoroughly rinsed in an ice-cold saline solution (0.9% NaCl, pH 7.0), and the mucosa of these intestinal segments was scraped off using a glass slide onto the ice. All tissue samples were collected at the same site of relevant tissue in all the animals, cleaned thoroughly of adhering tissues, quickly frozen and stored individually in plastic bags at −80 °C until they were analyzed.

### 2.3. Sample Analysis

#### 2.3.1. Chemical Composition of Feed

Feed samples (meadow hay, concentrate, concentrate + Zn and Hmix) were analyzed in triplicate for dry matter (No. 967.03), nitrogen (No. 968.06), crude protein (No. 990.03), ash (No. 942.05), fat (No. 983.23), acid-detergent fiber and neutral-detergent fiber contents according to standard procedures [24,25], as previously described [22]. The bioactive compounds of the Hmix presented in Table 1 were analyzed by ultra-high-resolution mass spectrometry using a Dionex UltiMate 3000RS system (Thermo Scientific, Darmstadt, Germany) with a charged aerosol detector connected to a high-resolution quadrupole time-of-flight mass spectrometer (HR/Q-TOF/MS, Impact II, Bruker Daltonic GmbH, Bremen, Germany) [22].

#### 2.3.2. Preparation of Tissue Homogenates

For the determination of antioxidant parameters, samples from each tissue were separated into three parts. Each tissue piece was weighed into a test tube and homogenized separately using a homogenizer (Polytron^®^ PT 1600 E, Kinematica AG, Switzerland). One part was homogenized in a cold 50 mM phosphate buffer (pH 7.0) containing 1 mM EDTA to give a 5% homogenate (*w*/*v*). After centrifugation at 10,000× *g* for 20 min at 4 °C (Boeco U-320 R, Hamburg, Germany), the supernatants were separated and used for the analysis of glutathione peroxidase (GPx) activity, catalase (CAT) activity, and total antioxidant capacity (TAC). For determination of superoxide dismutase (SOD) and Cu/Zn SOD activity, homogenization was carried out in an ice-cold 10 mM TRIS buffer containing 0.25 M sucrose (pH 7.4) to make a 10% homogenate (*w*/*v*). The homogenization was performed at full speed for 30 s for 8 cycles. The homogenates were then centrifuged at 10,000× *g* for 30 min at 4 °C, and the resulting supernatant was used for enzyme assays. The last portion of the tissue sample, used for estimating lipid oxidation products by means of the TBARS (thiobarbituric acid reactive substances) method, was homogenized for 15 s at high speed in deionized distilled water (DDW) to make a 10% homogenate (*w*/*v*), and butylated hydroxytoluene (BHT, 7.2%) dissolved in ethanol (95%) was added before homogenization [26]. 

#### 2.3.3. Antioxidant Enzyme Activity Assays

Total superoxide dismutase (SOD, EC 1.15.1.1) activity was determined by spectrophotometric assay according to the procedure of Marklund and Marklund [27]. This method is based on the ability of SOD to inhibit the pyrogallol autoxidation at alkaline pH. Briefly, an aliquot of tissue extract was mixed with Tris-cacodylic buffer (50 mM, pH 8.2) containing diethylenetriamine pentaacetic acid (1 mM) and a pyrogallol solution (5 mM) and subsequently the absorbance was monitored at 420 nm for 3 min. using a UV/visible spectrophotometer (Shimadzu UV-2550, Kyoto, Japan). The activity was expressed as the amount of enzyme that inhibits the oxidation of pyrogallol by 50%, which is equal to 1 unit per mg of protein. The activity of Cu/Zn SOD was assayed by inhibition of cytosolic SOD with 1 mM KCN under the same experimental conditions.

The activity of glutathione peroxidase (GPx, EC 1.11.1.9) in tissues was performed by measuring the oxidation of NADPH according to Paglia and Valentine [28]. The assays were carried out at 25 °C and hydrogen peroxide was used as the substrate. The absorbance was read at a wavelength of 340 nm and enzymatic activity was presented as U/g protein. One unit of GPx activity was defined as the amount of sample needed to oxidize 1 µmol of NADPH per min at 25 °C. The activity of blood GPx and hemoglobin (Hb) content was analyzed using commercial kits from Randox, UK. 

The activity of catalase (CAT, EC 1.11.1.6) was determined according to the method of Aebi [29], which involves monitoring the rate of disappearance of H_2_O_2_ in the presence of tissue homogenate at 240 nm. The tissue extract was reacted with freshly prepared 10 mM H_2_O_2_ in 50 mM phosphate buffer (pH 7.0). One unit of CAT activity is defined as the decomposition of 1 µmol of H_2_O_2_ per minute at room temperature. Results are expressed in units per mg of tissue protein.

#### 2.3.4. Lipid Oxidation and Total Antioxidant Capacity Determination

A modified fluorometric TBARS method was used to determine lipid oxidation in the serum and tissues, as described by Jo and Ahn [26]. Samples (250 µL) of serum or tissue homogenates were mixed with 100 µL of sodium dodecyl sulfate (8.1%), 750 µL of HCl (0.5 M), 750 µL of thiobarbituric acid (TBA, 20 mM), 25 µL of BHT (7.2 %) and 250 µL of DDW in test tubes and incubated for 15 min in a water bath at 90 °C. After cooling for 10 min, 2.5 mL of n-butanol/pyridine solution (15:1, *v*/*v*) and 0.5 mL of DDW was added and the samples were centrifuged at 3000× *g* for 15 min. The fluorescence of the upper layer was read at 520-nm excitation and 550-nm emission in a fluorometer (Shimadzu RF-1501, Kyoto, Japan). A standard curve was prepared using 1,1,3,3-tetramethoxypropane (malondialdehyde-bis), and the extent of lipid peroxidation was expressed as µmol of malondialdehyde (MDA) formed per L of serum or nmol MDA per g of tissue protein.

The total antioxidant capacities (TAC) of the serum, liver, kidney and intestinal mucosa (supernatant obtained as described above) were performed by ferric reducing antioxidant power (FRAP) assay using the method of Benzie and Strain [30], with some modifications. The Fe^3+^ reduction rate of the tissue sample was measured in a FRAP reagent prepared by mixing 0.3 M acetate buffer with pH 3.6, 10 mM 2,4,6-tris(2-pyridyl)-*s*-triazine and 20 mM FeCl_3_.6H_2_O (10:1:1) at 37 °C and 593 nm. Absorbance was measured after 30 minutes and the results were calibrated with the FRAP value of the standard (FeSO_4_.7H_2_O) and expressed in µmol Fe^2+^ per g of tissue protein or mmol Fe^2+^ per liter of sera. Total protein in the tissue homogenates was assayed by the method of Bradford [31], with bovine serum albumin as a standard. 

#### 2.3.5. Trace Elements Measurement

All tissue and feed samples were dried (105 °C for 48 h), ground and digested with a concentrated HNO_3_ (65%) and H_2_O_2_ (30%) mixture (3:1) using closed pressure vessels in a MWS 4 Speedwave microwave (Berghof Company, Eningen, Germany). The serum samples were only diluted with 0.05% Triton X-100 solution [32]. Trace element (Zn, Cu, Fe and Mn) concentrations were analyzed by flame atomic absorption spectrometry in an air-acetylene flame using a double beam atomic absorption spectrophotometer (AA-7000 Series, Shimadzu Co., Kyoto, Japan) with deuterium background correction. The certificate reference material of lyophilized human plasma ClinCheck Control (Recipe, Munich, Germany), bovine liver BCR-185R and pig kidney ERM-BB186 (Institute for Reference Materials and Measurements, Geel, Belgium) were routinely run in each analysis to verify the precision of the analysis. Mineral concentration in all tissue samples and dietary components was expressed as mg/kg DM and in serum as mg/L.

#### 2.3.6. Statistical Analysis

All statistical analyses were performing using the GraphPad Prism software (GraphPad Prism version 8.4.2., GraphPad Software, San Diego, CA, USA). Differences between diets with and without additives were analyzed by two-way analysis of variance (ANOVA). Experimental data were analyzed as a 2 × 2 factorial in a randomized complete block design representing two main factors: Hmix (with and without) and zinc (with and without), followed by the Tukey post-hoc test for pairwise multiple comparisons, where appropriate. The Least Significant Difference test (Fisher’s LSD) was applied post-hoc to determine significant differences among the treatments in case of a significant interaction (Hmix × Zn). For each analysis, the individual lamb was considered as an experimental unit. The data presented are the mean values and pooled standard errors of the mean (SEM). Differences were considered significant when *p* ≤ 0.05.

## 3. Results

### 3.1. Antioxidant Enzyme Activity 

The data showing the antioxidant enzyme activities in tissues of infected lambs are presented in Table 2. No effect of Hmix and/or Zn supplementation on the liver total SOD activity was detected, while the Hmix × Zn interaction (*p* = 0.014) affected Cu/Zn SOD activity in the liver, with increased activity in the Hmix group compared to CON (*p* < 0.01). The activity of both SODs did not change in the kidney tissue, but a tendency towards increased SOD activities (SOD, *p* = 0.052; Cu/Zn SOD, *p* = 0.063) in the Hmix treatments was determined.

The effect of Zn intake was observed in the activity of total SOD and Cu/Zn SOD (*p* = 0.002, *p* = 0.001, respectively) in the duodenal mucosa, with the highest activity recorded in lambs fed Zn alone over those fed the CON diet. Neither the total SOD nor Cu/Zn SOD activity in jejunal and ileal mucosa was influenced by the experimental diets.

An interaction of Hmix × Zn was identified for GPx activity in the liver (*p* = 0.004), duodenal mucosa (*p* < 0.001) and blood (*p* = 0.020), and increased GPx activity was found in lambs fed diets with Hmix or Zn alone as compared to those receiving the CON diet or the Hmix and Zn combination (Table 2 and Table 4). GPx activity in the kidney, jejunal and ileal mucosa was not affected by the treatment.

Feeding the Hmix diets significantly increased CAT activity in the kidney (*p* = 0.025) and ileal mucosa (*p* = 0.05), while no effect of dietary treatments on CAT activity in the liver, duodenal, and jejunal mucosa was observed.

### 3.2. Lipid Oxidation and Total Antioxidant Capacity

As shown in Table 3, there were no effects of Hmix and Zn supplementation or interaction between feed additives on lipid oxidation in the tissues. The kidney tissue of lambs fed the diets supplemented with Zn showed a tendency towards lower MDA values (*p* = 0.055). There was an interaction between Hmix and Zn supplementation (*p* = 0.01) on lipid oxidation in the serum (Table 4). The MDA values were decreased in serum of lambs fed diets with Hmix (*p* < 0.01) or Zn (*p* < 0.05) alone or their combination (*p* < 0.05) as compared to those receiving the non-supplemented diet. 

The total antioxidant capacity of the liver (*p* = 0.049) and the duodenal mucosa (*p* = 0.011) was affected by Hmix intake. Duodenal TAC was also significantly affected by the Hmix × Zn interaction (*p* = 0.020) and was significantly increased in all supplemented groups compared with the CON group. Antioxidant capacity of the ileal mucosa was affected by both the Hmix (*p* = 0.004) and Zn (*p* < 0.001), with the highest values occuring in lambs fed a combination of Hmix and Zn compared to the CON group (*p* < 0.001) and both experimental treatments (*p* < 0.05). No significant effects on the TAC of serum, kidney, and jejunal mucosa were observed due to treatment (Table 3 and Table 4).

### 3.3. Mineral Profile

Serum Zn concentration was affected by Zn supplementation *(p* = 0.012), with the highest Zn level occurring in the Hmix + Zn treatment compared to the CON group (*p* < 0.05). There were no effects of Hmix and Zn supplements on the levels of Cu and Fe in the serum (Table 4). 

Mineral (Zn, Mn, Cu, Fe) concentrations in tissues are presented in Table 5. Intake of the diets enriched with Zn increased the concentration of Zn in the liver, with the highest values found in lambs fed diets with the Hmix and Zn combination over those fed the Hmix alone (*p* < 0.05). The Zn concentration in the other tissues of the lambs was not affected by the dietary treatment. 

An interaction (*p* = 0.024) between Hmix and Zn was recorded for the Cu concentration in the kidney, with an increased Cu level in lambs fed diets with Zn alone in comparison with those fed the CON diet (*p* < 0.01) or the one supplemented with the Hmix and Zn combination (*p* < 0.05). The dietary inclusion of Zn resulted in an elevated Cu concentration in the duodenal mucosa (*p* = 0.039). The Cu concentrations in the liver and intestinal mucosa of the jejunum and ileum were not influenced by the Zn and Hmix treatment. 

In the duodenal mucosa, there was observed a Hmix × Zn interaction (*p* = 0.004) for the concentration of Fe, with increased values in lambs fed the diet with Zn alone compared to those receiving the Hmix and Zn combination (*p* < 0.01). Other tissue Fe concentrations were not changed by the dietary treatment. The Mn concentration in tissues did not differ among the dietary treatments; however, there was a trend of increased Mn level in the kidneys of lambs fed the Zn diets (*p* = 0.063).

## 4. Discussion

Our previous study in lambs infected with the gastrointestinal nematode *H. contortus* indicate the anthelmintic potential of the medicinal herbs and zinc due to a reduction in the fecal egg count and abomasal worm burden; in addition, the clinical signs of haemonchosis, such as anemia, were improved by treatment. However, no significant effects on body weight and mean live-weight gain of infected lambs were observed throughout the experiment due to Hmix and/or Zn treatment [22].

The occurrence of oxidative stress in lambs experimentally infected by *H. contortus* has been documented mainly due to the detection of lipid peroxidation, decreased GPx activity and TAC in plasma or serum [2,33] as well as a decreased level of reduced glutathione and SOD activity in erythrocytes [34] or elevated plasma lactate dehydrogenase level [35]. Data on the antioxidant status in tissues such as liver, kidney and intestinal mucosa of nematode-infected lambs treated with herbal nutraceuticals and organic Zn are currently lacking.

Zn is a co-factor for the enzymatic activity of cytosolic Cu/Zn superoxide dismutase (Cu/Zn-SOD), but in this study dietary Zn supplementation did not affect the activity of this Zn-containing metalloenzyme in tissues, except for the duodenal mucosa. In contrast, Pal et al. [36] reported a positive correlation between plasma and liver Cu/Zn SOD activity and Zn levels in ewes fed an organic mineral source. Approximately 75–85% of all plasma zinc is bound to albumin, which acts as a transport protein for essential metal ions to allow their systemic distribution [37]. Serum albumin, which is a major Zn-carrier, is reduced in a host due to infection with the blood-sucking nematode *H. contortus* [38]. Therefore, zinc transport and distribution seem to be disturbed in lambs suffering from nematode infection and may influence Zn delivery to cells of target tissues for the synthesis of cytosolic zinc-containing Cu/Zn SOD. Supplementation of diets with Zn increased the activity of total SOD and Cu/Zn SOD only in the duodenal mucosa of lambs, even though duodenal Zn concentration was not affected by the treatment. On the other hand, Zn intake increased the duodenal Cu concentration; therefore, our results point out the relationship between Cu/Zn SOD activity and Cu level in the intestinal mucosa. Regarding the functioning of this antioxidant enzyme, it is important to consider that Cu and Zn work together, and actually, the ratio of both elements rather than the concentration of either of these trace elements helps the enzyme function properly [39]. 

Several mechanisms of flavonoids antioxidant activity have been described in vitro, including activation of antioxidant enzymes, direct scavenging of reactive oxygen species, metal chelating activity, inhibition of oxidases, reduction of α-tocopheryl radicals, increase in uric acid levels, and antioxidant properties of low molecular antioxidants [9]. The bioavailability of dietary phenolic compounds in ruminants is still controversial and their possible antioxidant mechanism in the animal tissues is unclear. Several studies suggest that only some dietary phenolic compounds are bioavailable in ruminants. Moñino et al. [40] observed several of the polyphenols (i.e., rosmarinic acid, carnosol and carnosic acid) in the muscle of lambs suckling from ewes fed with a rosemary-enriched concentrate, whereas, other phenols from rosemary were not detected in the lamb tissues. According to López-Andrés et al. [41], the improvement of the antioxidant capacity of liver and plasma in lambs fed with grass phenolic compounds was not related to the direct transfer of phenolic compounds from herbage to the animal tissues. Dietary polyphenols undergo extensive biotransformation in the small intestine and in hepatic metabolism; therefore, their antioxidant effects might be expected within the gastrointestinal tract, where polyphenols may come into direct contact with cells before subsequent absorption and metabolism [10]. In our study, the intake of Hmix affected the catalase activity in the ileal mucosa and kidney. Moreover, feed supplementation with Hmix alone resulted in higher Cu/Zn SOD activity in the liver as well as increased GPx activity in the duodenal mucosa and blood when compared to unsupplemented lambs. Although tissue lipid peroxidation was not affected by dietary treatment, lower MDA values than in the control lambs were observed in the serum of all supplemented groups. Polyphenols could indirectly upregulate the antioxidant defense system, probably via activation of various transcription factors, including nuclear factor NrF2 (nuclear factor erythroid 2-related factor) and NF-κB (nuclear factor kappa B) [42,43]. Our previous study [44] showed a strong positive correlation between the total polyphenols content and antioxidant capacity of some of the medicinal plants (wormwood, chamomile, fumitory and mallow) and the dietary substrate containing a mix of these herbs possessed strong ruminal antioxidant capacity. Furthermore, strong antioxidant activity has been documented for the biologically active compounds that were the most abundant in the Hmix, such as quercetin, luteolin, carnosic acid, carnosol and rosmarinic acid [22,45,46,47] and probably contributed significantly to the antioxidant effect of the Hmix. It was reported that quercetin treatment can increase the activity of antioxidant enzymes (GPx, SOD, CAT, and glutathione reductase) and reduce lipid peroxidation in tissues in vivo, suggesting that the flavonoid quercetin enhances the antioxidant defense system [48,49]. For our future studies, determining the bioaccessibility of the main bioactive compounds found in herb mixture as well as their potential bioactivity and antioxidative effects ought to be addressed.

To further assess the antioxidant potential of both dietary supplements, the total antioxidant capacity of serum and tissues was determined in this experiment. Higher TAC values were observed in the liver, duodenal and ileal mucosa of lambs fed Hmix, and a similar pattern was found in the intestinal mucosa of lambs supplemented with Zn. The antioxidants that react in the FRAP assay include ascorbic acid, α-tocopherol, uric acid, bilirubin, and polyphenolic compounds, such as catechins and other flavonoids [50]. Based on the assay principle as well as the mechanism of polyphenols antioxidant action mentioned above, we assume that increased TAC in the tissues of animals treated with Hmix can likely be associated with the ability of bioactive polyphenolic compounds of herbs to increase non-enzymatic antioxidant levels. Similarly, zinc induces the synthesis of antioxidants, such as metallothioneins (MTs), which can protect cells against oxidative damage, and also influences glutathione formation by affecting the expression of glutamate–cysteine ligase [11,14]. Therefore, Zn acts directly on the neutralization of free radicals by glutathione or indirectly as a glutathione peroxidase cofactor [14]. Intake of the diet with the inclusion of either Zn or Hmix alone increased GPx activity in the blood, liver and duodenal mucosa when compared with control lambs or those fed the diet containing both Hmix and Zn. The results of the present study indicate that in terms of the antioxidant activity of GPx, the sole intake of Hmix or Zn may offer greater potential for the elimination of reactive oxygen metabolites than a combination of both additives.

Various studies have been carried out in animals [51,52] and cell culture systems [53] to determine the effect of bioactive plant compounds on mineral absorption. Stef and Gergen [51] reported generally moderate or poor correlation between total phenols and the accumulation of minerals (Zn, Cu, Fe, Mn) in the liver and muscles of chickens receiving medicinal herbs rich in a different type of polyphenols (flavonoids, phenol acids, benzoic acid derivate, phenylpropanoids derivate, and condensed tannins). Supplementation of the diet with polyphenol-rich plant products for 4 weeks had a marginal effect on the status of Zn, Fe, and Cu in piglets with a nutritionally adequate supply of those microelements [52]. Afsana et al. [54] demonstrated no effect of tannic acid supplementation on Zn, Cu, and Mn absorption in rats, but Fe absorption was reduced. There is great variability in the extent to which polyphenol-metal chelation may affect mineral bioavailability. It has been shown that the content and type of polyphenols present in the feed may have a specific influence on the accumulation of microelements in tissue [51]. In the present work, the tissue concentration of Zn, Cu, Fe and Mn was not affected by the intake of Hmix, indicating that medicinal plants containing flavonoids (54%), diterpenes (27%), and phenolic acids (19%) had no negative effect on microelements uptake in the tissues and serum of infected lambs.

Feed supplementation with organic Zn increased serum and liver levels of Zn, which play a crucial role in host immune response to a nematode infection [3,55]. Schafer et al. [3] reported a reduction of Cu and Zn levels in the liver of lambs infected with *H. contortus*; however, no significant decrease was observed in experimentally infected lamb parenterally treated with a combination of Zn and Cu. Gastrointestinal nematodes in sheep interfere with copper metabolism by causing a pH increase in the abomasal and duodenal digesta [56]. However, it seems that the mechanism of parasite interference with copper by blocking its absorption from the gastrointestinal tract is not the only mechanism by which the parasitism negatively affects Cu metabolism in the sheep [12]. In the present study, Zn supplementation increased Cu content in the duodenal mucosa and also in the kidney tissue. It is important to note the competition between copper and zinc for binding to a common transporter in enterocytes; however, this occurs especially when elevated levels of Zn are present in the diet [57]. Iskandar et al. [58] reported that improved Cu status in rats supplemented with a moderately high level of Zn correlated with increased duodenal mRNA expression of several Zn- trafficking proteins (i.e., MT-1, ZnT-1, ZnT-2, and ZnT-4). Therefore, it is possible that increased expression of these Zn-trafficking proteins may alter the distribution of Zn within absorptive enterocytes in a manner that alleviates the block in the activity of a Cu transporter. The results of the present study showed that Zn from an organic source (Zn chelate of glycine hydrate) added to the diet at the level up to the maximum authorized Zn content in complete feed (120 mg Zn/kg) [59] did not interfere with Cu absorption in the duodenal mucosa. The use of organic Zn sources rather than inorganic salts may improve the mineral supply due to their higher bioavailability, mainly because of their different route of absorption and lower interaction with other minerals and dietary components in the gastrointestinal tract [57]. Our finding is in line with the results of an experiment carried out on rabbits and chicken, which showed that the antagonism between Zn and Cu was not found when organic Zn was added to the diets [60,61].

Zinc status has a marked impact on intestinal iron absorption, metabolism and modulation of Fe homeostasis. With adequate Zn dietary intake, intestinal iron absorption and transcellular transport are stimulated via induction of divalent metal transporter-1 (DMT1) and ferroportin (FPN) expression, respectively [62]. Moreover, regulation of Fe absorption and mobilization by zinc might help to keep the cellular redox status in balance, because iron is known as a pro-oxidant while zinc is seen as an antioxidant [63]. Several dietary factors, such as polyphenols, have been shown to affect iron absorption [64]. In our study, Fe concentration in the duodenal mucosa was significantly affected by the interaction and was elevated in lambs supplemented with Zn alone compared to those fed the combination of Zn and medicinal herbs. Dietary polyphenols possess antioxidant properties, including chelation of metals, such as iron; for this reason, it is important to study whether the regular intake of bioactive polyphenolic compounds may impair iron absorption and homeostasis. Flavonoids can affect iron status by modulating the expression and activity of proteins involved in the systemic regulation of iron metabolism and uptake [64]. Several studies have established that bioactive dietary polyphenolic compounds significantly reduce iron absorption in the duodenum, but the precise mechanism of action is unknown [53,65,66]. Lesjak et al. [66] reported that quercetin treatment can influence intestinal iron uptake by inhibition of iron absorption and by decreasing the expression of duodenal DMT1 and FPN. However, Mazhar et al. [67] showed that various polyphenols regulate ferroportin expression and iron homeostasis differently, which might result from differences in their structures. Furthermore, it is required to elucidate the precise mechanism of action of herbal bioactive compounds on mineral absorption in order to improve the efficient utilization of trace elements from various sources (organic/inorganic) in ruminants with a gastrointestinal infection.

## 5. Conclusions

In conclusion, the inclusion of medicinal herbs into the diets of infected lambs influenced their antioxidant status due to increasing the kidney and ileal catalase activity, while zinc supplementation elevated the duodenal superoxide dismutase activity. Dietary intake of both additives alone or in combination positively affected the total antioxidant capacity of tissues. The present study showed that herbal supplementation did not significantly influence the tissue uptake of trace elements (Zn, Cu, Fe, and Mn). Feeding diets enriched with organic zinc improved the serum and liver zinc levels and increased copper concentration in the duodenal mucosa of lambs with nematode infection. Our results indicate that feed supplementation with medicinal herbs containing bioactive compounds and organic zinc may attenuate the adverse effects of parasite infection by stimulating endogenous antioxidant defense systems of small ruminants.

## Figures and Tables

**Table 1:** Chemical composition of the meadow hay, concentrates and herbal mix.

Analyzed Composition	MH	C	C + Zn	Hmix
Dry matter (g/kg)	900	878	876	905
Neutral-detergent fiber (g/kg DM)	651	136	254	532
Acid detergent fiber (g/kg DM)	556	83	93	452
Crude protein (g/kg DM)	163	309	352	207
Nitrogen (g/kg DM)	27	49	56	33
Ash (g/kg DM)	91	29	30	84
Fat (g/kg DM)	21	13	12	26
**Microelements Content (mg/kg DM)**				
Zinc	45.1	25.4	86.0	26.4
Iron	147.0	68.3	69.8	414.3
Copper	7.0	7.1	8.1	9.9
Manganese	77.7	24.1	25.8	45.1
**Phytochemical Content (g/kg DM)**				
Phenolic acids	n.d.	n.d.	n.d.	3.55
Flavonoids	n.d.	n.d.	n.d.	9.96
Diterpenes	n.d.	n.d.	n.d.	4.89

MH: Meadow hay, C: Concentrate, C + Zn: Concentrate and zinc chelate of glycine hydrate, Hmix: Herbal mix, DM: Dry matter, n.d.: Not determined.

**Table 2:** The effect of dietary supplementation with the herbal mixture and/or organic zinc on the activity of antioxidant enzymes in the tissues of infected lambs (*n* = 6).

Enzyme Activity	Dietary Treatment				SEM	*p*-Value		
	CON	Hmix	Zn	Hmix + Zn		Hmix	Zn	Hmix × Zn
**Total SOD (U/mg protein)**								
Liver	58.23	71.31	67.60	65.98	2.035	0.148	0.601	0.068
Kidney	42.20	47.57	46.44	48.19	0.918	0.052	0.173	0.304
Duodenum	5.87 ^a^	6.19 ^ab^	8.49 ^b^	7.81 ^ab^	0.366	0.771	**0.002**	0.423
Jejunum	5.82	7.10	7.13	7.45	0.264	0.119	0.106	0.337
Ileum	4.92	6.39	5.88	5.98	0.236	0.093	0.545	0.141
**Cu/Zn SOD (U/mg protein)**								
Liver	50.16 ^A^	66.76 ^B^	59.77 ^AB^	57.34 ^AB^	2.065	0.060	0.979	**0.014**
Kidney	31.83	36.64	34.96	35.73	0.681	0.063	0.443	0.170
Duodenum	3.52 ^a^	3.68 ^a^	5.36 ^b^	4.58 ^ab^	0.231	0.403	**0.001**	0.221
Jejunum	3.32	4.34	4.50	4.60	0.206	0.151	0.072	0.240
Ileum	3.23	3.90	3.46	3.38	0.136	0.280	0.592	0.173
**GPx (U/g protein)**								
Liver	27.96 ^AB^	31.00 ^B^	32.90 ^B^	24.90 ^A^	1.013	0.159	0.734	**0.004**
Kidney	37.83	42.22	39.95	39.78	1.111	0.366	0.945	0.331
Duodenum	27.35 ^A^	32.02 ^B^	32.90 ^B^	28.54 ^A^	0.636	0.862	0.258	**<0.001**
Jejunum	25.26	26.88	26.09	23.72	0.792	0.818	0.479	0.233
Ileum	25.53	27.96	26.44	28.00	0.955	0.328	0.814	0.830
**CAT (U/mg protein)**								
Liver	45.70	54.92	56.50	58.70	2.672	0.293	0.184	0.514
Kidney	53.86	58.17	51.89	66.46	2.162	**0.025**	0.428	0.203
Duodenum	2.41	2.61	2.50	1.82	0.124	0.296	0.139	0.067
Jejunum	1.55	1.76	1.87	1.96	0.093	0.414	0.181	0.761
Ileum	1.59	1.63	1.30	1.65	0.054	**0.050**	0.172	0.127

CON: Control, Hmix: Herbal mix, Zn: Zinc chelate of glycine hydrate, SOD: Total superoxide dismutase activity, Cu/Zn SOD: Cu/Zn Superoxide dismutase, GPx: Glutathione peroxidase activity, CAT: Catalase. ^a,b^ Means with different superscript letters in a row are significantly different (*p* < 0.05) using Tukey’s post hoc test. ^A,B^ Means with different superscript letters in a row are significantly different (*p* < 0.05) using Fisher’s Least Significant Difference (LSD) post hoc test. Bold values denote statistical significance at *p* < 0.05.

**Table 3:** The effect of dietary supplementation with the herbal mixture and/or organic zinc on lipid oxidation and total antioxidant capacity in the tissues of infected lambs (*n* = 6).

Antioxidant Parameters	Dietary Treatment				SEM	*p*-Value		
	CON	Hmix	Zn	Hmix + Zn		Hmix	Zn	Hmix × Zn
**MDA (nmol/g protein)**								
Liver	73.48	72.01	77.32	74.50	3.403	0.770	0.666	0.927
Kidney	55.08	48.91	47.09	44.30	1.663	0.164	0.055	0.590
Duodenum	48.38	43.11	48.03	45.47	1.971	0.353	0.809	0.745
Jejunum	82.30	72.39	84.40	91.39	3.806	0.849	0.179	0.278
Ileum	66.52	65.13	67.95	64.61	1.751	0.532	0.904	0.796
**TAC (µmol/g protein)**								
Liver	46.73	50.47	49.00	54.93	1.243	**0.049**	0.160	0.639
Kidney	41.31	40.58	39.08	45.97	1.276	0.229	0.531	0.140
Duodenum	23.02 ^A^	27.30 ^B^	25.46 ^B^	25.68 ^B^	0.492	**0.011**	0.617	**0.020**
Jejunum	24.23	27.45	27.25	27.88	0.564	0.074	0.108	0.220
Ileum	24.03 ^a^	25.40 ^ab^	27.44 ^b^	30.04 ^c^	0.553	**0.004**	**<0.001**	0.329

CON: Control, Hmix: Herbal mix, Zn: Zinc chelate of glycine hydrate, MDA: Malondialdehyde, TAC: Total antioxidant capacity. ^a,b,c^ Means with different superscript letters in a row are significantly different (*p* < 0.05) using Tukey’s post hoc test. ^A,B^ Means with different superscript letters in a row are significantly different (*p* < 0.05) using Fisher’s Least Significant Difference (LSD) post hoc test. Bold values denote statistical significance at *p* < 0.05.

**Table 4:** Antioxidant activity and microelements (Zn, Cu, Fe) concentration in serum of infected lambs supplemented with the herbal mixture and/or organic zinc (*n* = 6).

Serum Parameters	Dietary Treatment				SEM	*p*-Value		
	CON	Hmix	Zn	Hmix + Zn		Hmix	Zn	Hmix × Zn
GPx (U/g Hb)	381.08 ^A^	464.21 ^B^	476.86 ^B^	419.44 ^AB^	15.162	0.648	0.369	**0.020**
MDA (µmol/L)	0.279 ^B^	0.187 ^A^	0.203 ^A^	0.221 ^A^	0.011	0.074	0.298	**0.010**
TAC (mmol/L)	0.336	0.349	0.341	0.355	0.005	0.199	0.579	0.921
Zinc (mg/L)	0.740 ^a^	0.810 ^ab^	0.850 ^ab^	0.903 ^b^	0.021	0.109	**0.012**	0.823
Copper (mg/L)	0.815	0.893	0.857	0.845	0.037	0.675	0.967	0.572
Iron (mg/L)	1.418	1.940	1.858	1.920	0.107	0.180	0.329	0.286

CON: Control, Hmix: Herbal mix, Zn: Zinc chelate of glycine hydrate, GPx: Glutathione peroxidase activity, Hb: Hemoglobin, MDA: Malondialdehyde, TAC: Total antioxidant capacity. ^a,b^ Means with different superscript letters in a row are significantly different (*p* < 0.05) using Tukey’s post hoc test. ^A,B^ Means with different superscript letters in a row are significantly different (*p* < 0.05) using Fisher’s Least Significant Difference (LSD) post hoc test. Bold values denote statistical significance at *p* < 0.05.

**Table 5:** Microelements concentration in the tissues of infected lambs supplemented with the herbal mixture and/or organic zinc (*n* = 6).

Mineral Concentration	Dietary Treatment				SEM	*p*-Value		
	CON	Hmix	Zn	Hmix + Zn		Hmix	Zn	Hmix × Zn
**Zinc (mg/kg DM)**								
Liver	118.34 ^ab^	102.32 ^a^	122.84 ^ab^	129.20 ^b^	3.653	0.471	**0.026**	0.104
Kidney	105.23	106.42	104.16	106.16	1.481	0.617	0.835	0.897
Duodenum	98.61	97.72	96.27	98.54	0.700	0.635	0.599	0.282
Jejunum	98.48	96.76	98.18	100.35	1.687	0.950	0.647	0.589
Ileum	98.26	98.51	95.93	97.94	0.824	0.512	0.400	0.609
**Copper (mg/kg DM)**								
Liver	295.88	350.36	295.90	322.67	17.420	0.270	0.704	0.704
Kidney	16.44 ^A^	17.41 ^AB^	18.56 ^B^	17.06 ^A^	0.286	0.613	0.099	**0.024**
Duodenum	7.03	6.84	8.08	7.64	0.220	0.462	**0.039**	0.777
Jejunum	7.44	9.04	8.15	8.04	0.242	0.116	0.749	0.074
Ileum	11.71	12.20	11.85	11.81	0.182	0.563	0.751	0.493
**Iron (mg/kg DM)**								
Liver	81.62	93.78	87.40	94.11	2.810	0.106	0.591	0.631
Kidney	104.28	107.23	103.06	107.70	2.671	0.505	0.948	0.881
Duodenum	143.38 ^AB^	178.28 ^AB^	215.41 ^B^	103.97 ^A^	13.395	0.109	0.961	**0.004**
Jejunum	59.54	65.22	64.16	64.76	1.713	0.383	0.561	0.480
Ileum	56.96	64.29	61.45	61.22	1.352	0.195	0.792	0.168
**Manganese (mg/kg DM)**								
Liver	11.43	10.35	11.48	11.42	0.294	0.347	0.354	0.403
Kidney	4.12	4.26	4.46	4.53	0.078	0.495	0.063	0.819
Duodenum	22.81	19.24	17.40	18.44	1.071	0.555	0.155	0.286
Jejunum	6.08	6.57	5.59	6.22	0.225	0.231	0.361	0.874
Ileum	7.33	7.80	6.53	7.98	0.263	0.071	0.544	0.345

CON: Control, Hmix: Herbal mix, Zn: Zinc chelate of glycine hydrate, DM: Dry matter. ^a,b^ Means with different superscript letters in a row are significantly different (*p* < 0.05) using Tukey’s post hoc test. ^A,B^ Means with different superscript letters in a row are significantly different (*p* < 0.05) using Fisher’s Least Significant Difference (LSD) post hoc test. Bold values denote statistical significance at *p* < 0.05.
